# Expression of Cartilage Developmental Genes in Hoxc8- and Hoxd4-Transgenic Mice

**DOI:** 10.1371/journal.pone.0008978

**Published:** 2010-02-02

**Authors:** Claudia Kruger, Claudia Kappen

**Affiliations:** 1 Department of Genetics, Cell Biology, and Anatomy, University of Nebraska Medical Center, Omaha, Nebraska, United States of America; 2 Center for Human Molecular Genetics, Munroe-Meyer Institute for Genetics and Rehabilitation, University of Nebraska Medical Center, Omaha, Nebraska, United States of America; University of Western Ontario, Canada

## Abstract

Hox genes encode transcription factors, which regulate skeletal patterning and chondrocyte differentiation during the development of cartilage, the precursor to mature bone. Overexpression of the homeobox transcription factors Hoxc8 and Hoxd4 causes severe cartilage defects due to delay in cartilage maturation. Matrix metalloproteinases (MMPs), bone morphogenetic proteins (BMPs) and fibroblastic growth factors (FGFs) are known to play important roles in skeletal development and endochondral bone formation and remodeling. In order to investigate whether these molecules are aberrantly expressed in Hoxc8- and/or Hoxd4-transgenic cartilage, we performed quantitative RT-PCR on chondrocytes from Hox-transgenic mice. Gene expression levels of Bmp4, Fgf8, Fgf10, Mmp9, Mmp13, Nos3, Timp3, Wnt3a and Wnt5a were altered in Hoxc8-transgenic chondrocytes, and Fgfr3, Ihh, Mmp8, and Wnt3a expression levels were altered in Hoxd4-transgenic chondrocytes, respectively. Notably, Wnt3a expression was elevated in Hoxc8- and reduced in Hoxd4-transgenic cartilage. These results suggest that both transcription factors affect cartilage maturation through different molecular mechanisms, and provide the basis for future studies into the role of these genes and possible interactions in pathogenesis of cartilage defects in Hoxc8- and Hoxd4-transgenic mice.

## Introduction

Hox genes encode transcription factors that are involved in patterning the individual elements of the developing skeleton during the development of cartilage, the precursor to mature bone. Studies on animals provide evidence that patterning, growth and differentiation of skeletal elements are affected by mutations in Hox genes. Later in the process of endochondral ossification, as cartilage is replaced by bone, patterning defects and abnormal differentiation manifest in skeletal anomalies and growth defects [1].

Using a binary transgenic system [2], we have previously shown that overexpression of the homeobox transcription factors Hoxc8 and Hoxd4 results in severe cartilage defects [3], [4], characterized by delayed maturation, reduced proteoglycan content, accumulation of immature chondrocytes and decreased maturation to hypertrophy. Vertebral and rib cartilages contain accumulation of proliferating chondrocytes, indicating that cartilage maturation is affected by overexpression of Hoxc8 and Hoxd4, respectively. The cartilage of the ribs is weak and structurally insufficient, resulting in pulmonary failure and perinatal death [3], [4].

Earlier results demonstrated that the cartilage defects induced by overexpression of Hoxd4 could be rescued by supplementation of the micronutrient folate during pregnancy. When maternal diets were supplemented with folic acid, transgenic offspring were found to have less severe Hoxd4-induced skeletal defects. Alcian Blue staining of cartilage in ribs and vertebral column was restored by folate supplementation, and rigidity of the skeleton was improved [3]. Since folate is required for growth and differentiation of chondrocytes, the beneficial effect of folate in Hoxd4-transgenic mice might indicate a local deficiency in folate utilization that would result from deregulated expression of genes encoding folate transport proteins or folate metabolic enzymes. Our extensive qRT-PCR analyses on Hoxc8- and Hoxd4- transgenic animals revealed no changes in mRNA expression pattern of genes and enzymes involved in nucleotide synthesis, protein methylation and DNA methylation related to the folate metabolic pathway [5]. These findings suggested that mechanisms other than the folate pathway genes themselves are altered in the transgenic cartilage, and that other cellular or molecular pathways respond to folate supplementation during cartilage formation.

Our current understanding of chondrogenesis, the earliest phase of skeletal development, is based on studies in chicken and mice, as well as knockouts. The formation of most bones in the trunk skeleton of a vertebrate embryo occurs through endochondral ossification. In this process, a cartilage model is formed that is then converted into bone through the action of osteoblasts. This process involves mesenchymal cell condensation and chondrogenic differentiation. Chondrocytes undergo stages of proliferation, prehypertrophy and finally mature to hypertrophy. Blood vessels and osteoprogenitor cells invade the cartilage model, which leads to the formation of trabecular bone. The sequence of this well-defined order of steps is very important for proper cartilage and bone formation. Any disruption of this process, such as through overexpression of the Hoxc8- or Hoxd4-transgenes in our experimental paradigms, is expected to involve genes that control the progression of chondrocyte differentiation or the cellular phenotype of chondrocytes.

This hypothesis led us to further investigate the expression of genes known to be involved in skeletal development, such as the transcription factors Sox9, Sox5 and Sox6 [6], [7], local regulators of cartilage differentiation like fibroblast growth factors, hedgehog proteins, bone morphogenetic proteins, and their respective receptors [8], as well as Wnt signaling pathway components. We also included Prl1, a molecule that we had previously identified in a differential display screen as potentially regulated by Hoxc8 [9] and effectors in cartilage development, such as the Metalloproteinases and Inhibitors. Our choices were further guided by finding that the majority of the 37 genes were either not represented or not consistently detectable in DNA microarray assays of Hox transgenic cartilage (Kruger et al., manuscript in preparation).

## Methods

### Transgenic Mice

Transgenic mice were created by the VP16-dependent binary system [2]; phenotypes and similarities of defects in Hoxd4- and Hoxc8-transgenic mice have been characterized and published [3], [4]. The binary transgenic system is based on the potent transcriptional activator VP16 of Herpes Simplex Virus. One line, the transactivator (TA), harbors the transgene encoding VP16 under the control of a developmentally regulated promoter from the Hoxc8 gene. The other line, the transresponder (TR), harbors a Hox transgene under the control of an immediate early promoter of HSV. Activation of the immediate early promoter of the transresponder transgene requires the presence of VP16 protein. Crosses of TA and TR for the Hoxd4-transgene result in two genotypes (TA/+ +/+ and TA/+ TR/+), while the Hoxc8-transgene crosses produce four genotypes (TA/+ +/+, TA/TA +/+, TA/+ TR/+, and TA/TA TR/+). In the results presented here, we identify only two genotypes: the control genotype (TA) containing at least one TA and no TR, and the experimental genotype (TA+TR) containing at least one TA and one TR. DNA isolated from tails of individual animals was used for genotyping by semi-quantitative PCR [4], [10].

### Preparation of Primary Chondrocytes from Transgenic Mice

The dissection of rib cages, at 18.5 days of gestation, and purification of chondrocytes were done exactly as described earlier [5], [11]. Cells were transferred into Trizol reagent (Invitrogen, Carlsbad, CA, USA) and RNA was extracted as described in Kruger et al. (2006) [5]. Complementary DNA was obtained by reverse transcription (1^st^ Strand Synthesis System for RT-PCR, Invitrogen) from maximally 5 µg RNA of each sample, following the supplier's instructions. Purification of cDNA was carried out using QIAquick® PCR purification columns (Qiagen, Valencia, CA, USA). RNA as well as cDNA concentrations were measured with a NanoDrop® ND-1000 Spectrophotometer (NanoDrop Technologies, Inc., Rockland, DE, USA).

### Primers

Primers for amplification were designed according to the parameters described in Kruger et al. (2006) [5]. Primers for the gene encoding Glyceraldehyde phosphate dehydrogenase (Gapdh) were used as provided by Applied Biosystems (Foster City, CA). The locations and sequences of primers are listed in Table 1. Where possible, the expected product amplicon was designed to span an exon/exon junction to avoid amplification from potentially contaminating genomic DNA. The ENSEMBLE genome browser contains the transcript and exon information for the genes investigated here (accession numbers are given in Table 1).

**Table 1:** Genes investigated in this study: primer location, sequences and amplification efficiency.

Symbol	Full name (Alternative abbreviation)	Accession#	Forward primer Position	Reverse primer Position	Exon-Exon Boundary?	Forward primer - Sequence	Reverse primer - Sequence	Amplification rate	Number of samples
Bmp4	Bone morphogenetic protein 4	NM_007554	738–758	810–832	yes	CGAGCCAACACTGTGAGGAGT	AGGTTGAAGAGGAAACGAAAAGC	1.81	44
Bmpr1a	Bmp receptor, type 1A	NM_009758	1720–1743	1848–1833	yes	GGAAATGGCTCGTCGTTGTATTAC	GGCCGCAAGCGTTTCA	1.94	22
Bmpr1b	Bmp receptor, type 1B	NM_007560	1369–1392	1452–1433	yes	AAGAAAAATGGAACTTGCTGCATA	GGGTGGGATGTCAACCTCAT	1.86	22
Bmpr2	Bmp receptor, type 2	NM_007561	1368–1391	1509–1488	yes	CAGAGAGAAGCAGAGACCCAAGTT	CATCCTCTCCTCAGCACACTGT	1.94	22
Ctnnb1	Catenin beta 1, ß-catenin	NM_007614	1851–1866	1958–1940	yes	ACCCAACGGCGCACCT	CCGAGCAAGGATGTGGAGA	2.07	22
Cbf-	Core binding factor beta	NM_022309	544–565	627–602	yes	CTAGCCGGGAATATGTCGACTT	TAACACACACTCCATTCAGAATCATG	1.49	21
Col2a1	Procollagen, type II, alpha 1	NM_031163	273–298	348–327	no	AATGGGCAGAGGTATAAAGATAAGGA	CATTCCCAGTGTCACACACACA	1.57	44
Ext1	Exostoses (multiple) 1	NM_010162	1645–1665	1647–1629	yes	CGGTTTCTGCCCTATGACAAC	GCCATACGGTGAAGGCAAA	1.89	21
Fgf8	Fibroblast growth factor 8	NM_010205	701–718	772–752	yes	GCCAAGAGCAACGGCAAA	CAGCGCCGTGTAGTTGTTCTC	1.90	22
Fgf10	Fibroblast growth factor 10	NM_008002	672–696	755–729	yes	CCGTACAGTGTCCTGGAGATAACAT	CATGGCTAAGTAATAGTTGCTGTTGAT	1.82	22
Fgf18	Fibroblast growth factor 18	NM_008005	407–426	515–492	yes	GGGAGTCAAGTCCGGATCAA	TGAACACGCACTCCTTGCTAGTAC	1.66	22
Fgfr1	Fibroblast growth factor receptor 1	NM_010206	2104–2126	2205–2186	yes	GTGTGGTCTTTTGGAGTGCTCTT	ACCCTCCTTCAGCAGCTTGA	1.81	33
Fgfr2	Fibroblast growth factor receptor 2	NM_010207	2834–2857	2919–2898	yes	ACTGCACCAATGAACTGTACATGA	TTCGACCAACTGCTTGAATGTG	1.63	22
Fgfr3	Fibroblast growth factor receptor 3	NM_008010	1815–1836	1910–1892	yes	ACCGAGGACAATGTGATGAAGA	AGGTAGCCGGCCATTTGTG	1.65	33
Fgfr4	Fibroblast growth factor receptor 4	NM_008011	2467–2488	2606–2586	yes	AAACTGCCCCTCAGAGCTGTAT	GGCGGAGGTCAAGGTACTCTT	1.90	22
Gapdh	Glyceraldehyde-3-phosphatase dehydrogenase	NM_008084	649–669	751–733	no	CCAGAACATCATCCCTGCATC	GGTAGGAACACGGAAGGCC	1.83	107
Hoxc8	Homeobox c8	NM_010466	610–631	725–702	yes	CGAAGGACAAGGCCACTTAAAT	AGGTCTGATACCGGCTGTAAGTTT	1.84	62
Hoxd4	Homeobox d4	NM_010469	1658–1678	1748–1722	yes	TTCGGTGAACCCCAACTACAC	AAATTCCTTTTCCAGTTCTAGGACTTG	1.33	45
Ihh	Indian hedgehog protein precursor	NM_010544	683–702	747–732	no	CCCCAACTACAATCCCGACA	TCATGAGGCGGTCGGC	1.58	22
Lrp5	Low density lipoprotein receptor-related protein 5	NM_008513	4049–4064	4175–4153	yes	CTGCGACGGTGAGGCC	GAAGGAGTCACACTGTTGCTTGA	1.88	11
Lrp6	Low density lipoprotein receptor-related protein 6	NM_008514	4461–4482	4578–4554	yes	TTCCAACAGTCCTTCCACACAT	GCTAGGAGCATAGTCACTGTCACAG	1.56	33
Mmp3	Matrix metalloproteinase 3	NM_010809	1308–1332	1426–1401	yes	GGAGGTTTGATGAGAAGAAACAATC	GTAGAGAAACCCAAATGCTTCAAAGA	1.34	33
Mmp8	Matrix metalloproteinase 8	NM_008611	901–919	1005–986	yes	GCACACCCAAAGCCTGTGA	GAGGATGCCGTCTCCAGAAG	1.72	52
Mmp9	Matrix metalloproteinase 9	NM_013599	1195–1215	1268–1248	yes	CCAAGGGTACAGCCTGTTCCT	GCACGCTGGAATGATCTAAGC	1.86	63
Mmp13	Matrix metalloproteinase 13	NM_008607	478–501	595–575	yes	AATCTATGATGGCACTGCTGACAT	GTTTGGTCCAGGAGGAAAAGC	1.73	50
Nos3	Nitric-oxide synthase 3, endothelial	NM_008713	3425–3443	3489–3471	yes	ATGGAGCTGGATGAAGCCG	TCCTCGTGGTAGCGTTGCT	1.81	22
Pfn1	Profilin 1	NM_011072	282–302	382–362	yes	ATTACGCCAGCTGAGGTTGGT	TCCCGGATCACAGAACATTTC	1.51	32
Prl1	Protein tyrosine phosphatase 4a1 (Ptp4a1)	NM_011200	1222–1239	1295–1272	no	GGGTGCCTGATGCCATTG	CACATTGGGTAATATGCATGACAA	1.81	35
Pthlh	Parathyroid hormone-like peptide (Pthrp)	NM_008970	277–299	362–341	yes	AGTTAGAGGCGCTGATTCCTACA	GGACACTCCACTGCTGAACCA	1.69	31
Runx2	Runt related transcription factor 2	NM_009820	496–518	573–547	yes	CAAGTAGCCAGGTTCAACGATCT	GACTGTTATGGTCAAGGTGAAACTCTT	1.82	33
Runx3	Runt related transcription factor 3	NM_019732	958–977	1083–1059	yes	CGCTCACAATCACCGTGTTC	CCTTGGTCTGGTCTTCTATCTTCTG	1.86	32
Sox5	SRY-box containing gene 5	NM_011444	1654–1673	1798–1784	yes	ATGGTGTGGGCGAAAGATGA	GGCGGGCCTGCTCCT	1.49	19
Sox6	SRY-box containing gene 6	NM_011445	2083–2104	2202–2183	yes	AATTCTTCAGGCCTTCCCTGAC	CTTAGCCGGGCCTGTTCTTC	1.47	19
Sox8	SRY-box containing gene 8	NM_011447	477–598	679–658	yes	GCTTGCTGAGTGAAAGCGAGAA	CGCCTTGGCTGGTATTTGTAAT	1.94	43
Sox9	SRY-box containing gene 9	NM_011448	731–750	805–787	yes	GCAGACCAGTACCCGCATCT	CTCGTTCAGCAGCCTCCAG	1.83	44
Tcfap2a	Transcription factor AP-2, alpha	NM_011547	340–360	479–461	yes	GCCGATCCATGAAAATGCTTT	GGCGCTGGTGTAGGGAGAT	1.66	21
Timp3	Tissue inhibitor of Mmp3	NM_011595	2175–2195	2264–2244	no	GGGTGCCCTTCACTTAATTGC	CAACTGCCCCTTTCATGAGAA	1.67	33
Wdr5	WD repeat domain 5	NM_080848	217–236	357–340	yes	AGCACAGCCCACTCCTTCCT	GCCAACCATTCCCCATTG	1.83	22
Wnt3a	Wingless-related MMTV integration site 3A	NM_009522	399–418	493–474	yes	TGGCCCTGTTCTGGACAAAG	CTGCACAGGAGCGTGTCACT	1.63	18
Wnt5a	Wingless-related MMTV integration site 5A	NM_009524	999–1019	1073–1058	yes	TTCTGTCTTTGGCAGGGTGAT	ACCCCAGCTGCGCTCA	1.61	22

Messenger RNA sequences for the investigated genes were taken from GenBank (accession numbers). The positions of primer sequences are listed as annotated in ENSEMBL, Mouse genome version 52 (as of December 2008). The number of samples used for calculation of the primer-pair-specific amplification rate is given in the last column.

### Quantitative Real-Time PCR

Using the ABI Prism 7000 Instrument (Applied Biosystems, Foster City, CA, USA), gene expression was evaluated in chondrocyte preparations from individual mice belonging to several independent families of Hoxc8- and Hoxd4-transgenic mice and their non-transgenic littermates. Each PCR reaction (25 µl) was performed on 4 ng of cDNA template in 12 µl of water, 0.25 µl of each primer solution (10 µM) and 12.5 µl of SYBR Green Master Mix (Applied Biosystems). The thermal cycling reaction starts with 2 minutes at 50°C and 10 minutes at 95°C for optimal DNA polymerase activation. The PCR reactions consisted of a denaturation step of 15 seconds at 95°C, annealing and extension for one minute at 60°C, for a total of 40 cycles.

Analysis was performed with ABI Prism 7000 SDS Software Version 1.0. Measurements were done in triplicates and obtained values were averaged. The Gapdh gene was chosen as a reference gene for normalization, since its expression levels are comparable between control and transgenic samples; comparisons to other commonly used reference genes, such a cyclophilin or Polε4 confirmed the uniformity of expression levels in different experimental groups for the Gapdh reference. Applying the formula C_Tgene_−C_TGapdh_ = ΔC_T_ normalized each gene to measurements for Gapdh cDNA in the same sample. Comparison of transgenic samples to non-transgenic littermate controls was achieved in a second subtraction, which yielded the ΔΔC_T_ values: ΔΔC_T_ = ΔC_Ttransgenic_−ΔC_Tcontrol_. Amplification efficiencies were determined for each gene-specific reaction from the slope of the linear portion of the amplification reaction. The efficiencies and amplification rates shown in Table 1 were calculated as previously described [5] from at least 11 independent samples for each gene. The amplification of the target gene and the endogenous control occurred in separate tubes. To use the comparative C_T_ method, we ascertained that the efficiencies of the target and endogenous control amplifications were approximately equal. To calculate the fold-change of expression levels, we averaged ΔC_T_ values for transgenic and control groups, respectively (± SEM). Thus, graphs representing fold-change data do not contain standard deviations. To estimate the “relative fold-change”, we used the formula f = r^ΔΔCT^ (absolute ΔΔC_T_ value), with r representing amplification rate (r = amplification efficiency e+1).

### Statistical Evaluation

For statistical analyses, we used the Data Analysis Pack module implemented in Microsoft Excel X as well as GraphPad PRISM® (GraphPad Software, San Diego, USA). Two-tailed Student's T-tests with 95% confidence intervals were performed to analyze differences in gene expression between the controls and TR-containing transgenic mice. Coefficients for the correlation between Hox gene expression levels and expression levels of investigated genes (Table S1) were calculated based on ΔC_T_ values. The correlation coefficient r is dimensionless and ranges from −1.0 to 1.0, with −1.0 representing a strong negative, and 1.0 a strong positive linear relationship. We set an arbitrary cut-off at |0.6| for considering an r-value as correlated.

## Results

In order to determine whether expression of genes in the chondrocyte differentiation and maturation pathways was altered in Hoxc8- or Hoxd4-transgenic mice, we assayed the prevalence of transcripts for genes known to participate in regulation of the chondrocyte differentiation pathway. Quantitative real-time PCR was performed on cDNA samples derived from RNA isolated from primary chondrocytes of individual rib cartilages from Hoxc8- and Hoxd4-transgenic mice, respectively. In earlier published work [3], [4], [10], we demonstrated that the Hoxc8- and Hoxd4-transgenes are specifically activated in chondrocytes in our transgenic mice. Both transgenes are reproducibly overexpressed in respective transgenic cells prior to birth [5]. In the samples used for the present study, Hoxc8 expression levels were 5.29-fold higher in Hoxc8-transgenic primary rib chondrocytes compared to their littermate controls, and Hoxd4 expression levels were 17.22-fold higher in Hoxd4-transgenic cartilage, compared to their littermate controls. The magnitude of Hoxd4 transgene expression appears higher than for the Hoxc8 transgene because there is virtually no Hoxd4 expressed in non-transgenic rib chondrocytes, while Hoxc8 is normally expressed in ribs.

**Figure 1:**
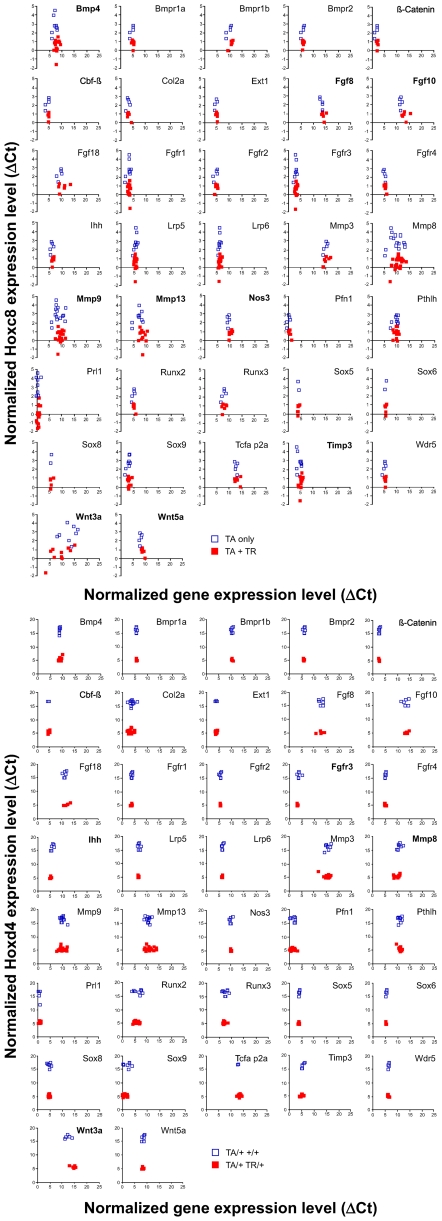
Gene expression in Hoxc8- and Hoxd4-transgenic chondrocytes. Quantitative real-time PCR was performed in triplicate on cDNA prepared from mRNA that was isolated from primary rib chondrocytes. Rib cages were dissected at 18.5 days of gestation from individual Hoxc8- (**A**) and Hoxd4- (**B**) transgenic mouse embryos. Gapdh cDNA levels in each sample were used to standardize measurements. The results are plotted as Gapdh-normalized ΔC_T_ values for each gene relative to Gapdh-normalized Hoxc8 or Hoxd4 gene expression (ΔC_T_) in each sample. Low ΔC_T_ values reflect higher relative expression levels, and high ΔC_T_ values reflect low relative levels of gene expression. Each dot represents an individual animal (filled symbol  =  TR-containing samples, open symbol  =  controls). Bold panel labeling indicates statistical significant differences in expression levels between transgenic and control groups.

We investigated gene expression levels in at least 5 individual control and 5 TR-containing samples (from at least 3 families each) of our Hoxc8- and Hoxd4-transgenic lines, respectively (Table 2). The results are plotted as ΔC_T_ (expression level for each gene normalized to Gapdh) relative to the corresponding ΔC_T_ values for Hoxc8 or Hoxd4 gene expression the same sample. Each data point represents the average of triplicate measurements for an individual animal. Lower ΔC_T_ values indicate higher gene expression level; higher ΔC_T_ values correspond to lower expression level. TR containing animals (Figure 1A, 1B: red filled rectangles) always had a lower ΔC_T_ value for the expression of the Hoxc8- or Hoxd4-transgene compared to the respective littermate controls (Figure 1A, 1B: open white rectangles), clearly indicating higher levels of Hox gene expression in the respective transgenic samples. Low ΔC_T_ values, such as for Prl1, Sox9, Pfn1 and ß-Catenin indicate high overall expression levels, and conversely, high ΔC_T_ values for Fgf8, Fgf10, Mmp3, Tcfap2a, or Wnt3a indicate that expression of these genes is very low in primary chondrocytes under normal conditions. Genes with intermediate level of expression are Bmpr1a, Bmpr2, Ihh, Runx2, Runx3, Sox6 and Wdr5. Close clustering of data points for each gene along the X-axis dimension demonstrates low variability of measurements between individual animals.

**Table 2:** Gene expression in Hoxc8 and Hoxd4 transgenic animals normalized to Gapdh.

Gene	Control	Hoxc8-transgenic		Control	Hoxd4-transgenic	
	TA only	TA + TR	P-value	TA/+ +/+	TA/+ TR/+	P-value
**Bmp4**	**7.08±0.26 (n = 9)**	**7.83±0.19 (n = 13)**	**0.028**	8.61±0.06 (n = 9)	8.84±0.14 (n = 12)	0.220
Bmpr1a	4.41±0.36 (n = 5)	4.75±0.14 (n = 6)	0.356	5.43±0.08 (n = 6)	5.45±0.07 (n = 5)	0.834
Bmpr1b	9.65±0.45 (n = 5)	10.51±0.29 (n = 6)	0.131	10.19±0.18 (n = 6)	10.33±0.14 (n = 5)	0.584
Bmpr2	5.64±0.29 (n = 5)	5.76±0.11 (n = 6)	0.700	5.90±0.12 (n = 6)	5.76±0.06 (n = 5)	0.344
ß-Catenin	1.54±0.29 (n = 5)	1.97±0.09 (n = 6)	0.158	2.54±0.08 (n = 6)	2.45±0.05 (n = 5)	0.375
Cbf-ß	4.00±0.30 (n = 5)	4.51±0.07 (n = 6)	0.101	4.29±0.25 (n = 2)	4.57±0.09 (n = 8)	0.244
Col2a	2.87±0.29 (n = 5)	3.09±0.24 (n = 6)	0.574	3.55±0.25 (n = 14)	3.10±0.22 (n = 18)	0.185
Ext1	4.33±0.30 (n = 4)	4.85±0.12 (n = 6)	0.103	3.60±0.15 (n = 3)	3.65±0.09 (n = 8)	0.804
**Fgf8**	**13.18±0.23** **(n = 5)**	**14.05±0.27** **(n = 6)**	**0.039**	12.80±0.36 (n = 6)	12.64±0.56 (n = 5)	0.809
**Fgf10**	**11.55±0.19** **(n = 5)**	**13.20±0.54** **(n = 6)**	**0.026**	12.92±0.46 (n = 6)	13.53±0.29 (n = 5)	0.315
Fgf18	9.27±0.40 (n = 5)	10.45±0.74 (n = 6)	0.219	11.07±0.24 (n = 6)	11.99±0.47 (n = 5)	0.099
Fgfr1	2.87±0.27 (n = 9)	3.12±0.10 (n = 13)	0.336	3.83±0.09 (n = 6)	3.67±0.10 (n = 5)	0.293
Fgfr2	4.27±0.34 (n = 5)	4.64±0.19 (n = 6)	0.344	5.31±0.14 (n = 6)	5.29±0.04 (n = 5)	0.887
**Fgfr3**	2.89±0.16 (n = 8)	3.12±0.11 (n = 13)	0.238	**3.60±0.27** **(n = 6)**	**2.88±0.05** **(n = 5)**	**0.044**
Fgfr4	4.97±0.18 (n = 5)	5.20±0.16 (n = 6)	0.361	5.13±0.05 (n = 6)	4.94±0.15 (n = 5)	0.230
**Hoxc8**	**2.98±0.19 (n = 24)**	**0.24±0.15 (n = 38)**	**<0.001**			
**Hoxd4**				**15.53±0.51 (n = 21)**	**5.55±0.12 (n = 24)**	**p<0.001**
**Ihh**	5.69±0.25 (n = 5)	6.28±0.14 (n = 6)	0.059	**5.71±0.21** **(n = 6)**	**5.08±0.13** **(n = 5)**	**0.039**
Lrp5	5.84±0.21 (n = 9)	5.71±0.11 (n = 13)	0.550	6.63±0.18 (n = 6)	6.30±0.07 (n = 5)	0.148
Lrp6	5.94±0.12 (n = 9)	6.14±0.13 (n = 13)	0.275	6.33±0.11 (n = 6)	6.20±0.05 (n = 5)	0.332
Mmp3	14.77±0.36 (n = 5)	15.15±0.46 (n = 6)	0.551	15.48±0.28 (n = 9)	15.11±0.39 (n = 12)	0.480
**Mmp8**	9.43±0.66 (n = 15)	10.29±0.36 (n = 21)	0.231	**10.79±0.28** **(n = 8)**	**9.84±0.30 (n = 11)**	**0.038**
**Mmp9**	**8.51±0.46 (n = 15)**	**9.42±0.23 (n = 20)**	**0.007**	10.17±0.24 (n = 12)	10.02±0.28 (n = 20)	0.729
**Mmp13**	**7.06±0.54** **(n = 9)**	**8.71±0.29 (n = 13)**	**0.006**	10.32±0.24 (n = 12)	10.97±0.30 (n = 20)	0.143
**Nos3**	**9.48±0.16 (n = 5)**	**10.38±0.24** **(n = 6)**	**0.015**	9.81±0.23 (n = 6)	9.93±0.08 (n = 5)	0.661
Pfn1	−0.03±0.27 (n = 5)	0.36±0.20 (n = 6)	0.265	1.61±0.30 (n = 9)	1.34±0.0.27 (n = 13)	0.520
Prl1	0.37±0.20 (n = 8)	0.44±0.15 (n = 17)	0.878	0.20±0.14 (n = 8)	0.46±0.14 (n = 9)	0.219
Pthlh	9.45±0.38 (n = 9)	9.60±0.21 (n = 13)	0.720	10.87±0.21 (n = 9)	10.88±0.16 (n = 14)	0.975
Runx2	4.92±0.26 (n = 5)	5.24±0.15 (n = 6)	0.288	6.37±0.52 (n = 9)	5.59±0.28 (n = 13)	0.167
Runx3	7.44±0.37 (n = 5)	7.70±0.33 (n = 6)	0.624	7.31±0.33 (n = 9)	7.01±0.17 (n = 12)	0.392
Sox5	3.96±0.16 (n = 2)	3.96±0.11 (n = 7)	0.998	3.91±0.07 (n = 6)	3.75±0.10 (n = 5)	0.228
Sox6	5.43±0.27 (n = 2)	5.43±0.08 (n = 6)	0.981	5.22±0.10 (n = 6)	5.05±0.05 (n = 5)	0.183
Sox8	2.17±1.10 (n = 8)	3.43±0.78 (n = 13)	0.350	4.42±0.21 (n = 9)	4.41±0.07 (n = 13)	0.934
Sox9	2.77±0.25 (n = 8)	3.05±0.11 (n = 14)	0.264	1.80±0.43 (n = 9)	0.92±0.24 (n = 13)	0.070
Tcfap2a	12.68±0.23 (n = 5)	13.29±0.42 (n = 6)	0.101	12.77±0.10 (n = 2)	13.42±0.25 (n = 8)	0.244
**Timp3**	**4.41±0.28** **(n = 9)**	**5.06±0.16 (n = 13)**	**0.045**	5.32±0.19 (n = 6)	4.84±0.30 (n = 5)	0.199
Wdr5	4.89±0.28 (n = 5)	5.20±0.11 (n = 6)	0.301	6.30±0.07 (n = 6)	6.24±0.14 (n = 5)	0.678
**Wnt3a**	**12.85±1.05 (n = 8)**	**9.31±1.18 (n = 10)**	**0.052**	**12.3±0.47** **(n = 5)**	**14.42±0.44** **(n = 5)**	**0.011**
**Wnt5a**	**7.98±0.18** **(n = 5)**	**8.85±0.22** **(n = 6)**	**0.016**	8.40±0.19 (n = 6)	8.25±0.17 (n = 5)	0.565

ΔC_T_ values were determined for all controls (TA only) and TR-containing Hoxc8- and Hoxd4-transgenic samples. Means (± standard error of the mean) were calculated for all individuals tested in each group (n =  from 2 to 21) as indicated. P-values were obtained by performing two-tailed Student's T-tests.

### Gene Expression in Hoxc8-Transgenic Chondrocytes

**Figure 2:**
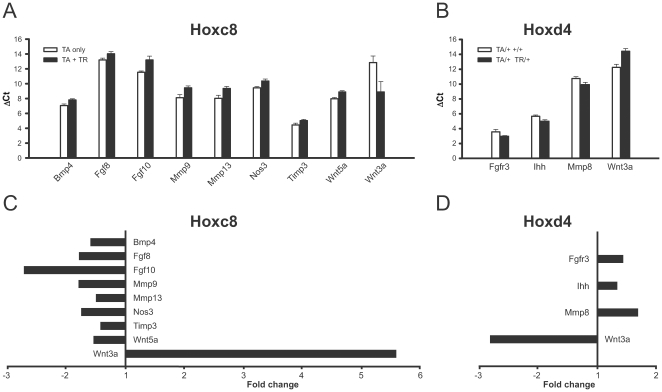
Altered gene expression levels in Hoxc8- and Hoxd4-transgenic cartilage. ΔC_T_ values (normalized to endogenous control Gapdh) were averaged for each investigated gene over the control group as well the Hoxc8-transgenic group (**A**,**C**) and the Hoxd4-transgenic group (**B**,**D**), respectively, and are plotted as mean ± standard error of the mean (SEM). Student's T-test was performed to confirm statistical significance (p<0.05). Higher ΔC_T_ values were found for Bmp4, Fgf8, Fgf10, Mmp9, Mmp13, Nos3, Timp3, and Wnt5a (**A**, filled bars) in Hoxc8-transgenic animals, indicating lower expression levels compared to littermate controls (**A**, open bars). Wnt3a expression was higher (lower ΔC_T_ value) in Hoxc-transgenic chondrocytes compared to controls. Lower ΔC_T_ values were found for Fgfr3, Ihh and Mmp8 (**B**, filled bars) in Hoxd4-transgenic animals, indicating higher expression levels compared to littermate controls (**B**, open bars). Wnt3a expression levels are lower in Hoxd4-transgenic chondrocytes (higher ΔC_T_ value) compared to the control group. Figures **C** and **D** present the relative fold-change using the comparative C_T_ method based on amplification efficiency for each gene (see Methods), respectively.

The comparison of chondrocyte pathway gene expression between Hoxc8-transgenic and control animals revealed significant differences (p<0.05) for the ΔC_T_ values of Bmp4, Fgf8, Fgf10, Mmp9, Mmp13, Nos3, Timp3, Wnt3a and Wnt5a (bold in Figure 1A). Except for Wnt3a, all these genes show decreased expression levels in transgenic chondrocytes compared to their littermate controls (Figure 2A). After normalizing to Gapdh, our endogenous reference gene, the fold-change was calculated using the comparative C_T_ method including the correction for amplification rate (Figure 2B). An increased level was found for Wnt3a with overall 5.6-fold (after amplification rate correction) elevated expression in transgenic samples compared to controls. The expression levels of Mmp13 and Fgf10 were more than 2-times lower in Hoxc8-transgenic animals compared to controls. Decreased expression levels of around 1.8-fold were found for Mmp9, Nos3 and Fgf8, and 1.5-fold lower expression for Bmp4 and Wnt5a (Figure 2B). A significant decrease of expression by 1.4-fold was observed for Timp3 in chondrocytes from Hoxc8-transgenic animals compared to controls. Thus, expression levels of Bmp4, Fgf8, Fgf10, Mmp9, Mmp13, Nos3, Timp3, Wnt3a and Wnt5a were significantly altered in primary chondrocytes from Hoxc8-transgenic mice.

### Gene Expression in Hoxd4-Transgenic Chondrocytes

In Hoxd4-transgenic chondrocytes, 33 of the 37 tested genes showed no significant differences in mRNA expression levels relative to controls, while 4 genes exhibited altered expression (Figure 2C). Wnt3a exhibited the greatest difference in ΔC_T_ values, in addition to Mmp8, Fgfr3 and Ihh, compared to controls (Figure 2D) (p<0.05). The expression levels of Wnt3a were 2.8-fold lower in Hoxd4-transgenic chondrocytes compared to controls. Mmp8 expression levels were increased 1.7-fold in transgenic animals compared to controls, and we found moderately (by 1.4- and 1.3-fold, respectively) but significantly elevated Fgfr3 and Ihh expression levels (Figure 2D). These results identify expression levels of Fgfr3, Ihh, Mmp8 and Wnt3a as significantly altered in primary chondrocytes from Hoxd4-transgenic mice.

### Relationship of Gene Expression to Transgene Levels

It remains to be investigated whether the differential gene expression levels in the cartilage of Hoxc8- and Hoxd4-transgenic animals are proximally causal to the cartilage defects, or whether they are distal indicators of abnormal differentiation caused by overexpression of the transcription factors. In order to address this question, we determined the correlation between gene expression levels and the levels of transgene expression in individual animals (data provided in Table S1). Our expectation was that for possible direct targets of the Hox transcription factors, their expression would be either strongly stimulated or repressed by the respective transgene, and hence, expression values would be expected to exhibit a strong positive or inverse correlation to transgene expression levels in the same animals. Meaningful correlations between expression of the respective gene and Hoxc8 were found for 20 genes: Positive correlation exists for Bmpr1b and Mmp3 in controls and Hoxc8-transgenic samples, and a consistent negative correlation was found for Fgfr4, indicating a potential repressive action of Hoxc8. However, neither of these genes alters expression levels in response to higher Hoxc8 levels (see Table 2) and they are thus unlikely to be directly regulated by Hoxc8. Strong correlations with Hoxc8 expression levels for Bmpr1a, Bmpr2, ß-Catenin, Cbf-ß, Ext1, Fgf8, Fgf18, Fgfr2, Runx3, Sox8, Sox9, Wdr5 and Wnt3a in controls are lost upon expression of the transgene, suggesting that transgene expression perturbs regulatory mechanisms for these genes. A gain of correlation of gene expression levels is detected for Nos3, Col2a and Wnt5a, with the latter two exhibiting an inverse relationship to Hoxc8 levels. However, Hoxc8 overexpression at the same time is correlated to lower levels of Nos3, excluding a direct link; in transgenic cells, the transcription factor interferes with Nos3 expression possibly through indirect mechanisms. A complex regulatory involvement for Hoxc8 in gene expression is indicated by the reversal by Hoxc8 overexpression in the direction of correlation for Profilin1 and Runx2 expression. Wnt5a expression is also inversely correlated to Hoxc8, suggesting a repressive relationship, and this is borne out by reduced Wnt5a levels in Hoxc8-transgenic chondrocytes.

For Hoxd4, the analysis indicates positive correlations for Fgfr4, Ihh, Sox5, Sox6 and Timp3, which are lost in the transgenic condition. For ß-Catenin and Wdr5, the strong correlation to Hoxd4 expression found in controls incurs reversal to inverse correlation in Hoxd4-transgenic samples; gain of a negative correlation in the transgenic condition was observed to Bmpr1a and Bmpr1b, and of a positive correlation for Fgf10 and Fgf18. Yet, there are no significant changes of expression level of these genes by transgene overexpression. Thus, for Hoxd4, this analysis suggests that the relationship between transcription factor levels and gene expression levels is complex.

## Discussion

Our data demonstrate that mRNA expression levels of particular genes are affected by overexpression of either Hoxc8 or Hoxd4 transcription factors in cartilage. These outcomes were measured in chondrocytes prepared on gestational day 18.5, just prior to birth. Thus, in order to interpret these results, we have to consider their relation to the process of cartilage formation during development.

Limited gene expression studies performed by quantitative real-time PCR on Hoxc8-transgenic rib chondrocytes were published by Cormier et al. (2003) [11], who compared gene expression levels in transgenic samples to FVB inbred mice as controls. Here, we compare gene expression levels from Hoxc8- (TA+TR) and Hoxd4-(TA/+ TR/+)-transgenic chondrocytes to controls (TA only and TA/+ +/+, respectively) from the same litter. In this way, any potential influence from the maternal environment should be excluded, since controls and experimental embryos were raised in the same dam. Differences in maternal effects between FVB control pregnancies and pregnancies from transgenic crosses might account for the differences of earlier results to the present study. Discrepancies might be further traced to sample preparation: in this study, RNA was extracted from single rib cages from animals at 18.5 days of gestation, whereas the earlier study prepared postnatal rib chondrocytes from newborns between birth and up to 2 days of age. In addition, the earlier study used poly-A+ enriched mRNA, and given the low expression levels of some of our test genes in chondrocytes, the generally low yield of this procedure may have introduced substantial variation. Finally, the earlier study used pools of cDNAs while the current measurements were done on individual samples. Because we find very small variations between technical replicates of the same sample, we feel confident about the reliability of the results presented here.

This comprehensive gene expression study on Hoxc8- and Hoxd4-transgenic primary chondrocytes revealed a few genes to be deregulated at the transcriptional level. Gene expression levels of eight genes (Bmp4, Fgf8, Fgf10, Mmp9, Mmp13, Nos3, Timp3, and Wnt5a) were significantly decreased in Hoxc8-transgenic animals, and Wnt3a expression was significantly increased. In Hoxd4-transgenic animals, elevated levels of Fgfr3, Ihh, Mmp8, and decreased levels of Wnt3a were detected. These results suggest that cartilage defects in Hoxc8- or Hoxd4-transgenic mice, while similar at the morphological level, may be based on different molecular mechanisms. They also identify these genes as plausible candidate targets for regulation by the respective Hox transcription factor.

### Bone Morphogenetic Proteins

Several genes are described in the literature that pattern distribution and proliferation of mesenchymal cells in condensation sites for future skeletal elements and therefore are important for limb and rib development [1], [12], [13]. During the events of chondrogenesis, Wnt, Fgf and Bmp signals are among the earliest signals [14]. BMPs, in addition to initiating chondroprogenitor cell determination and differentiation, also regulate later stages of chondrocyte maturation. Since Bmp4 is known to have a positive effect on hypertrophy [15], decreased expression level of Bmp4 in our Hoxc8-transgenic chondrocytes could be responsible for the observed delay in maturation in these cells [4]. Whether Hoxc8 itself directly deregulates BMP4 expression or whether other factors are involved warrants further investigation.

### Hedgehog Signaling

Ihh is involved in chondrocyte differentiation, proliferation and maturation during endochondral ossification. There is evidence that Ihh decreases the rate of progression to hypertrophy [16], [17]. Ihh is expressed in prehypertrophic chondrocytes and is involved in a negative feedback loop at the level of hypertrophic cells [18]. In this feedback scheme, Ihh stimulates synthesis of Parathyroid hormone-like hormone (Pthlh, formerly known as Pthrp) in the periarticular region of the growth plate, which then delays differentiation of proliferating chondrocytes into hypertrophic chondrocytes, which express Ihh mRNA [19]. Thus, chondrocyte hypertrophy is regulated through the interplay of Ihh and Pthlh signaling, and this regulation also involves Fgfs and Bmp signaling [8]. On the other hand, Ihh also has signaling functions independent of Pthlh [20]. Hoxd4-transgenic rib cage chondrocytes exhibit elevated Ihh expression compared to controls, but no change in Pthlh expression levels. Thus, for Hoxd4-transgenic chondrocytes, it is possible that elevated Ihh expression contributes to the observed phenotypes of delayed chondrocyte maturation and increased persistence of immature cells in the Hox transgenic cartilage, but likely this would be independent of the Pthlh feedback loop.

### Fibroblast Growth Factors

FGF signaling pathways are important regulators of chondrogenesis. Experimental studies in mice and cell culture show that signaling through Fgfr3 inhibits chondrocyte proliferation [21]. Mutations of this receptor cause a range of human bone disorders [22]. Targeted deletion of Fgfr3 in mice leads to increased regions of proliferating and hypertrophic chondrocytes [23], [24]. These findings at late gestational and postnatal stages of mouse development have led to the conclusion that Fgf signaling through this receptor is to limit chondrocyte proliferation and differentiation [25]. To date, only Fgf18 is discussed as a candidate for a ligand that signals through Fgfr3, based on the phenotype and expression pattern of Fgf18 null mice as well as on studies in vitro, where Fgf18 can stimulate the proliferation of cultured articular chondrocytes [26], [27].

In Hoxd4-transgenic chondrocytes, we found elevated gene expression of Fgfr3, while the Fgfs we investigated (Fgf8, Fgf10 and Fgf18) were not deregulated. Increased Fgfr3 signaling activity would be expected to further limit chondrocyte differentiation compared to controls, and thus could be responsible for the delayed chondrocyte maturation observed in the Hoxd4-transgenic animals. In contrast, in Hoxc8-transgenic chondrocytes, gene expression of Fgf8 and Fgf10 was found to be decreased, but no changes in expression levels of Fgf receptors were detected. Fgf8 signaling is required for the development of distal cartilage elements in the limb in chick embryos [28], indicating that Fgf8 promotes the process of proliferation and differentiation of mesenchymal cells into chondrocytes. Fgf10-deficient mice exhibit a complete truncation of fore and hind limbs [29], also supporting a role for Fgf10 in promoting chondrocyte proliferation, although in vitro studies of cultured murine cartilage cells from the ventral rib cage showed no discernable response to exogenously administered Fgf10 [30]. Nevertheless, the decrease in expression levels of Fgf8 and Fgf10 in Hoxc8-transgenic chondrocytes is consistent with a possible role in the phenotype we observe in these animals. It is interesting to note that the Fgf signaling pathway appears to be differentially affected by overexpression of Hoxc8 versus Hoxd4 transcription factors in chondrocytes, in that either the receptor or the growth factors are deregulated, but not both, by the same transgene. These results suggest that the two transcription factors, despite apparently similar phenotypic effects on cartilage formation, may regulate different transcriptional targets in chondrocytes or their progenitors.

### Matrixmetalloproteases and Tissue Inhibitor of Metalloproteases 3

A key process in endochondral ossification is the degradation of extracellular matrix (ECM) (for review, see Ortega et al. [31]). Matrixmetalloproteases (MMPs) are a large family of endopeptidases that are involved in degradation of many different components of the ECM, such as Collagen II, Collagen X and Aggrecan [32], [33]. MMPs are produced by a many different cell types, including epithelial cells, fibroblasts, inflammatory cells, and chondrocytes.

MMP13 (formerly known as Collagenase 3) is highly expressed during endochondral ossification; it is required for the transition from cartilage to bone at the growth plate of long bones [34], [35]. MMP13 actively degrades type II collagen, the major type of collagen in immature cartilage. During endochondral ossification, MMP13 is expressed in the lower zone of hypertrophic chondrocytes, co-localized with type X Collagen [36]. MMP9 (formerly known as Gelatinase B) is a key regulator of apoptosis of hypertrophic chondrocytes [37]. Although both MMPs are expressed in non-overlapping cell types, they act together in primary and secondary ossification. Gene expression levels of Mmp9 as well as Mmp13 were found significantly decreased in our Hoxc8-transgenic chondrocytes, which could lead to reduced ECM degradation. Additionally, MMP-mediated processes, such vascular invasion and chondrocyte apoptosis, might be disturbed or delayed based on lower expression levels of these genes. Reduced expression of Mmp9 and Mmp13 in Hoxc8-transgenic chondrocytes would be consistent with a delay in chondrocyte maturation and ossification, and thus might be responsible for the cartilage immaturity phenotype we observe in Hoxc8-transgenic mice.

In contrast, in Hoxd4-transgenic chondrocytes, we found Mmp8 expression to be elevated. Mmp8 (formerly known as Collagenase 2) is implicated in tissue remodeling under inflammatory conditions, and in wound healing [38], [39], [40]. Mmp8 was also reported to be expressed in mandibular and hind limb chondrocytes [41], and our results demonstrate it to be expressed in rib chondrocytes. The functional role of Mmp8 in chondrogenesis is currently unknown.

The activity of MMPs is regulated by the four members of the Tissue Inhibitor of Metalloproteinases (TIMP) family, which are mainly expressed by chondrocytes and bone-lining cells [42]. TIMP3 localizes to the ECM and inhibits MMP9, among other MMPs [43], [44]. Inhibition occurs when the active site of the MMPs becomes occupied in a 1:1 stoichiometric ratio by the TIMP protein, and a normal balance between MMPs and TIMPs is important for matrix remodeling and tissue architecture [45]. We found Timp3 expression decreased in Hoxc8-transgenic chondrocytes; it is intriguing to note that this coincides with decreased expression of Mmp9 and Mmp13. These results suggest that matrix maintenance and remodeling may be altered in Hoxc8-transgenic cartilage.

### Nitric Oxide Synthase 3

Nos3 encodes the murine endothelial Nitric oxide synthase, located on mouse chromosome 5 [46]. NO (nitric oxide) is known to mediate physiological responses in the nervous system, immune system and vascular smooth muscle. It has been reported that NO produced by eNOS and iNOS in osteoblasts and osteoclasts influences bone growth and remodeling [47], [48]. Studies in chick growth plate chondrocytes indicate that NO metabolism is required for development of the mature chondrocyte phenotype: it stimulates hypertrophy and seems to promote apoptosis of terminally differentiated chondrocytes [49]. NO also stimulates MMP production and activity in osteoarthritic joint cartilage [50]. The occurrence of limb reduction defects in Nos3-deficient mice [51] demonstrates a crucial role of NOS3 for normal bone development. Thus, it is tempting to speculate that lower expression of MMPs in Hoxc8-transgenic chondrocytes could be due to reduced NO production associated with lower Nos3 expression levels.

### ß-Catenin and Wnt Signaling

ß-Catenin plays important roles during skeletal development, and is the molecular node for signal transduction in the canonical Wnt signaling pathway [52]. Wnt-proteins are thought to bind to cell-surface receptors of the Frizzed family and activate Dishevelled family proteins; in turn, ß-Catenin becomes protected from proteasome-mediated degradation. Activated/de-phosphorylated ß-Catenin enters the nucleus and promotes the activation of target genes by interaction with TCF/LEF family of transcription factors. While in both Hoxc8- and Hoxd4-transgenic chondrocytes ß-Catenin expression was not altered at the transcriptional level, Wnt3a exhibited decreased expression levels in Hoxd4-transgenic rib cage chondrocytes. This reduced Wnt3a ligand expression and hence, reduced signaling, could be involved in delayed chondrocyte maturation in mice with Hoxd4 overexpression. On the other hand, we found Wnt3a expression levels elevated in Hoxc8-transgenic chondrocytes. Since Wnt3a promotes cell proliferation in many tissue systems, higher Wnt3a levels would be consistent with accumulation of proliferating cells in Hoxc8-transgenic cartilage. The most intriguing finding, however, is that Wnt3a levels are misregulated in opposite direction in Hoxc8- versus Hoxd4-transgenic chondrocytes, again providing evidence for the notion that both transgenes are involved in distinct pathways in the respective transgenic cartilage.

Wnt5a is expressed in proliferating and prehypertrophic chondrocytes and plays a pivotal role during chondrocyte proliferation and differentiation [53]. Chondrocyte differentiation was found delayed in developing long bones in Col2a1-Wnt5a transgenic mice, where Wnt5a is expressed in proliferating and immature chondrocytes under control the of the Col2a1 promoter [54]. Experiments in chicken also reveal a delay in maturation from proliferative to hypertrophic chondrocytes by overexpression of Wnt5a [55], [56], [57]. Long-term micromass cell cultures in vitro confirm that Wnt5a overexpression is associated with a delay in maturation of chondrocytes [58]. Wnt5a mouse mutants also show a delay in chondrocyte progression to hypertrophy, and as a consequence, limbs are shorter in Wnt5a^−/−^ mutants [54]. Thus, conditions of both overexpression and loss of Wnt5a resulted in altered proliferation of chondrocytes. We found Wnt5a expression decreased in Hoxc8-transgenic chondrocytes, consistent with a delay in maturation to hypertrophy. This, combined with the consistent negative correlation of Wnt5a expression to Hoxc8 expression levels, makes Wnt5a an excellent candidate for a transcriptional target of Hoxc8 in chondrocytes or their progenitors.

### Conclusions

By targeted investigation of 37 candidate genes, we found altered expression in transgenic cartilage of several genes that are known regulators of cartilage development. These genes could therefore be responsible for the cartilage defects observed in Hoxc8- and Hoxd4-transgenic animals, respectively. Strikingly, the subset of genes deregulated in Hoxc8-transgenic cartilage differs from the group of genes deregulated in Hoxd4-transgenic cartilage, indicating that different molecular pathways may be responsible for seemingly similar phenotypic outcomes, namely delayed chondrocyte proliferation and maturation. Nevertheless, our study identifies several genes that respond to altered Hoxc8 or Hoxd4 gene dosage, respectively, and thus might be targets of these transcription factors in chondrocytes or their progenitors. Further research will be needed to define the temporal sequence of changes in gene expression during chondrocyte differentiation, and to understand how Hox transcription factors and the deregulated genes we identified here interact to regulate chondrogenesis.

## Supporting Information

Table S1 Correlation between gene expression and transgene levels in Hoxc8- and Hoxd4-transgenic chondrocytes. The relationship of gene expression levels to transgene levels in individual animals was assessed using the Pearson's correlation coefficient (r) between ΔCT values, which are normalized to the reference gene. A correlation coefficient close to 1.0 indicates a strong positive relationship, r close to 0 indicates lack thereof, and negative r indicates an inverse relationship between transgene and candidate gene levels. Sample numbers smaller than 3 were excluded from consideration. Bold font indicates strong positive or negative correlation (r>|0.6|) of gene expression to transgene levels in control and/or transgenic individuals: black: strong correlation in controls and transgenics; green: lack of correlation in control but gain of strong correlation with expression of the respective transgene; red: strong correlation in controls but loss with expression of the respective transgene; blue: strong correlations in opposite directions in controls and transgenic samples.
(0.05 MB DOC)
