# Supplementary material for: Expression of Cartilage Developmental Genes in Hoxc8- and Hoxd4-Transgenic Mice
Source: PLoS One. 2010 Feb 2;5(2):e8978. doi: 10.1371/journal.pone.0008978 (PMC2814844; doi:10.1371/journal.pone.0008978)
Supplement: Table S1 — Correlation between gene expression and transgene levels in Hoxc8- and Hoxd4-transgenic chondrocytes. The relationship of gene expression levels to transgene levels in individual animals was assessed using the Pearson's correlation coefficient (r) between ΔCT values, which are normalized to the reference gene. A correlation coefficient close to 1.0 indicates a strong positive relationship, r close to 0 indicates lack thereof, and negative r indicates an inverse relationship between transgene and candidate gene levels. Sample numbers smaller than 3 were excluded from consideration. Bold font indicates strong positive or negative correlation (r>|0.6|) of gene expression to transgene levels in control and/or transgenic individuals: black: strong correlation in controls and transgenics; green: lack of correlation in control but gain of strong correlation with expression of the respective transgene; red: strong correlation in controls but loss with expression of the respective transgene; blue: strong correlations in opposite directions in controls and transgenic samples. (0.05 MB DOC) [file pone.0008978.s001.doc]

**Supplemental Information:**

**Correlation between gene expression and transgene levels in Hoxc8- and Hoxd4-transgenic chondrocytes.**

| **Gene** | **Hoxc8** | | **Hoxd4** | |
| --- | --- | --- | --- | --- |
|  | **TA only** | **TA + TR** | **TA/+ +/+** | **TA/+ TR/+** |
| Bmp4 | 0.15 (n=9) | 0.30 (n=13) | 0.42 (n=9) | 0.60 (n=12) |
| Bmpr1a | **0.87 (n=5)** | **0.28 (n=6)** | **0.05 (n=6)** | **-0.69 (n=5)** |
| Bmpr1b | **0.87 (n=5)** | **0.95 (n=6)** | **0.10 (n=6)** | **-0.63 (n=5)** |
| Bmpr2 | **0.90 (n=5)** | **0.31 (n=6)** | 0.02 (n=6) | -0.48 (n=5) |
| ß-Catenin | **0.90 (n=5)** | **-0.11 (n=6)** | **0.84 (n=6)** | **-0.69 (n=5)** |
| Cbf-ß | **0.91 (n=5)** | **0.10 (n=6)** | -1.00 (n=2) | 0.39 (n=8) |
| Col2a | **0.24 (n=5)** | **-0.76 (n=6)** | 0.17 (n=14) | 0.13 (n=18) |
| Ext1 | **0.77 (n=4)** | **-0.41 (n=6)** | -0.19 (n=3) | 0.26 (n=8) |
| Fgf8 | **-0.70 (n=5)** | **-0.14 (n=6)** | -0.11 (n=6) | 0.15 (n=5) |
| Fgf10 | 0.10 (n=5) | 0.09 (n=6) | **0.03 (n=6)** | **0.73 (n=5)** |
| Fgf18 | **0.68 (n=5)** | **0.34 (n=6)** | **0.43 (n=6)** | **0.90 (n=5)** |
| Fgfr1 | 0.58 (n=9) | -0.05 (n=13) | 0.30 (n=6) | 0.47 (n=5) |
| Fgfr2 | **0.79 (n=5)** | **0.33 (n=6)** | 0.32 (n=6) | -0.33 (n=5) |
| Fgfr3 | 0.49 (n=8) | 0.31 (n=13) | 0.07 (n=6) | -0.21 (n=5) |
| Fgfr4 | **-0.73 (n=5)** | **-0.68 (n=6)** | **0.86 (n=6)** | **-0.41 (n=5)** |
| Ihh | 0.52 (n=5) | 0.60 (n=6) | **0.65 (n=6)** | **0.05 (n=5)** |
| Lrp5 | 0.56 (n=9) | 0.00 (n=13) | -0.18 (n=6) | -0.29 (n=5) |
| Lrp6 | 0.50 (n=9) | 0.31 (n=13) | 0.52 (n=6) | 0.13 (n=5) |
| Mmp3 | **0.85 (n=5)** | **0.68 (n=6)** | 0.34 (n=9) | -0.59 (n=12) |
| Mmp8 | 0.04 (n=15) | 0.51 (n=21) | 0.35 (n=8) | 0.42 (n=11) |
| Mmp9 | 0.15 (n=15) | 0.20 (n=20) | -0.41 (n=12) | 0.11 (n=20) |
| Mmp13 | 0.49 (n=9) | -0.00 (n=13) | -0.14 (n=12) | -0.05 (n=20) |
| Nos3 | **0.30 (n=5)** | **0.65 (n=6)** | 0.41 (n=6) | -0.15 (n=5) |
| Pfn1 | **0.74 (n=5)** | **-0.84 (n=6)** | -0.35 (n=9) | -0.25 (n=13) |
| Prl1 | 0.30 (n=8) | 0.50 (n=17) | -0.46 (n=8) | 0.58 (n=9) |
| Pthlh | 0.51 (n=9) | 0.07 (n=13) | **0.15 (n=9)** | **-0.66 (n=14)** |
| Runx2 | **0.84 (n=5)** | **-0.93 (n=6)** | -0.36 (n=9) | -0.02 (n=13) |
| Runx3 | **0.77 (n=5)** | **0.39 (n=6)** | -0.10 (n=9) | -0.02 (n=12) |
| Sox5 | -1.00 (n=2) | 0.60 (n=7) | **0.78 (n=6)** | **0.19 (n=5)** |
| Sox6 | 1.00 (n=2) | 0.12 (n=6) | **0.76 (n=6)** | **-0.50 (n=5)** |
| Sox8 | **0.79 (n=8)** | **-0.28 (n=13)** | -0.36 (n=9) | -0.02 (n=13) |
| Sox9 | **0.83 (n=8)** | **0.06 (n=14)** | -0.29 (n=9) | -0.14 (n=13) |
| Tcfap2a | -0.42 (n=5) | -0.22 (n=6) | 1.00 (n=2) | 0.13 (n=8) |
| Timp3 | -0.20 (n=9) | 0.57 (n=13) | **0.84 (n=6)** | **0.44 (n=5)** |
| Wdr5 | **0.85 (n=5)** | **-0.18 (n=6)** | **0.64 (n=6)** | **-0.64 (n=5)** |
| Wnt3a | **0.16 (n=8)** | **0.66 (n=10)** | 0.10 (n=5) | -0.49 (n=5) |
| Wnt5a | **0.43 (n=5)** | **-0.89 (n=6)** | 0.48 (n=6) | -0.11 (n=5) |

Legend to Supplemental Information:

The relationship of gene expression levels to transgene levels in individual animals was assessed using the Pearson's correlation coefficient (r) between ∆C_T_ values, which are normalized to the reference gene. A correlation coefficient close to 1.0 indicates a strong positive relationship, r close to 0 indicates lack thereof, and negative r indicates an inverse relationship between transgene and candidate gene levels. Sample numbers smaller than 3 were excluded from consideration. **Bold font** indicates strong positive or negative correlation (r > |0.6|) of gene expression to transgene levels in control and/or transgenic individuals: **black**: strong correlation in controls and transgenics; **green**: lack of correlation in control but gain of strong correlation with expression of the respective transgene; **red**: strong correlation in controls but loss with expression of the respective transgene; **blue**: strong correlations in opposite directions in controls and transgenic samples.
